# Defect-Engineered Z-Scheme Heterojunction of Fe-MOFs/Bi_2_WO_6_ for Solar-Driven CO_2_ Conversion: Synergistic Surface Catalysis and Interfacial Charge Dynamics

**DOI:** 10.3390/nano15080618

**Published:** 2025-04-17

**Authors:** Ting Liu, Yun Wu, Hao Wang, Jichang Lu, Yongming Luo

**Affiliations:** 1 Faculty of Chemical Engineering, Kunming University of Science and Technology, Kunming 650500, China; liuting1@stu.kust.edu.cn (T.L.); 202210801211@stu.kust.edu.cn (Y.W.); 2 Key Laboratory of Yunnan Province for Synthesizing Sulfur-Containing Fine Chemicals, Kunming 650500, China; lujichangc7@kust.edu.cn; 3 The Innovation Team for Volatile Organic Compounds Pollutants Control and Resource Utilization of Yunnan Province, Kunming 650500, China; 4 Faculty of Environmental Science and Engineering, Kunming University of Science and Technology, Kunming 650500, China

**Keywords:** photocatalytic CO_2_ reduction, defect-engineered, heterojunctions, surface catalysis, charge transfer

## Abstract

The urgent need for sustainable CO_2_ conversion technologies has driven the development of advanced photocatalysts that harness solar energy. This study employs a CTAB-assisted solvothermal method to fabricate a Z-scheme heterojunction Fe-MOFs/V_O_-Bi_2_WO_6_ (FM/V_O_-BWO) for photocatalytic CO_2_ reduction. Positron annihilation lifetime spectroscopy (PALS) was employed to confirm the existence of oxygen vacancies, while spherical aberration-corrected transmission electron microscope (STEM) characterization verified the successful construction of heterointerfaces. X-ray absorption fine structure (XAFS) spectra confirmed that the defect configuration and heterostructure changed the surface chemical valence state. The optimized 1.0FM/V_O_-BWO composite demonstrated exceptional photocatalytic performance, achieving CO and CH_4_ yields of 60.48 and 4.3 μmol/g, respectively, under visible-light 11.8- and 1.5-fold enhancements over pristine Bi_2_WO_6_. The enhanced performance is attributed to oxygen vacancy-induced active sites facilitating CO₂ adsorption/activation. In situ molecular spectroscopy confirmed the formation of critical CO_2_-derived intermediates (COOH* and CHO*) through surface interactions involving four-coordinated and two-coordinated hydrogen-bonded water molecules. Furthermore, the accelerated interfacial charge transfer efficiency mediated by the Z-scheme heterojunction has been conclusively demonstrated. This work establishes a paradigm for defect-mediated heterojunction design, offering a sustainable route for solar fuel production.

## 1. Introduction

The persistent reliance on fossil fuels has resulted in a dual crisis of energy security and climate change, with anthropogenic CO_2_ emissions reaching critical thresholds that demand urgent technological interventions [1,2,3]. This urgency has galvanized research into photocatalytic CO_2_ valorization, a transformative paradigm that harnesses solar energy to convert greenhouse gases into renewable fuels (e.g., CO, CH_4_), thereby concurrently addressing energy deficits and carbon neutrality imperatives [4,5,6,7]. However, the thermodynamic and kinetic inertia of CO_2_ rooted in its robust linear geometry, high C=O bond dissociation energy (750 kJ·mol^−1^), and electron-deficient π-system demands photocatalysts capable of orchestrating multi-electron transfer processes while overcoming activation barriers under ambient conditions [8,9,10]. Although conventional semiconductor photocatalysts (e.g., TiO_2_, ZnO) exhibit promise in principle, their practical efficacy is crippled by intrinsic shortcomings: bandgaps misaligned with visible photons (>3.0 eV for TiO_2_), picosecond-scale electron-hole recombination, and non-selective surface reaction pathways that favor competitive H_2_ evolution over CO_2_ reduction [11,12,13]. These limitations underscore a critical materials science imperative: the rational design of heterostructured systems that synergistically integrate broadband light absorption, directional charge separation, and defect-engineered active sites to steer reaction specificity toward targeted solar fuel synthesis. In terms of inorganic materials, new photocatalyst materials based on bandgap engineering, nanodesign, Z-scheme, and multi-component systems are gradually designed and developed [14,15,16,17].

Recent breakthroughs in defect engineering and heterostructure architecture have revolutionized the rational design of high-performance photocatalytic systems, offering unprecedented control over charge carrier dynamics and surface reactivity. Bismuth-based semiconductors, exemplified by orthorhombic Bi_2_WO_6_, have attracted considerable interest due to their intrinsic visible-light absorption and unique layered configurations comprising alternating [Bi_2_O_2_]^2+^ and [WO_4_]^2−^ slabs, which theoretically facilitate anisotropic charge transportation [18,19,20]. However, their practical implementation in CO_2_ photoreduction is severely limited by suboptimal charge separation efficiency, primarily attributed to insufficient driving forces for redox reactions. To address these challenges, strategic defect engineering through oxygen vacancy incorporation has emerged as a transformative approach wherein OVs not only narrow the bandgap to enhance visible-light utilization but also generate localized electron-rich regions that strengthen CO_2_ chemisorption via Lewis acid–base interactions, thereby lowering the activation energy barrier for C=O bond cleavage [21]. Concurrently, the integration of metal–organic frameworks (MOFs), particularly iron-based Fe-MOFs featuring MIL-101 architectures, introduces a paradigm shift in interfacial charge management. These crystalline porous materials exhibit exceptional CO_2_ capture capacities due to their tunable pore geometries and open metal sites, while their semiconductor-like π-conjugated ligands enable synergistic band alignment with BWO for directed electron transfer [22,23,24]. Notably, pioneering work on Z-scheme heterojunctions (e.g., g-C_3_N_4_/UiO-66-NH_2_ composites [25]) has demonstrated that spatially separated redox reactions can be achieved through interfacial barriers, effectively suppressing backward recombination while maintaining strong redox potentials [26,27]. Nevertheless, the mechanistic interplay between crystallographic defects and interfacial charge equilibration in these hybrid systems remains poorly understood, particularly regarding how defect-mediated states influence carrier injection efficiency and photostability under prolonged irradiation.

Herein, we fabricated a novel Fe-MOFs/V_O_-BWO heterojunction photocatalyst via a CTAB-assisted in situ solvothermal method. The photocatalytic CO_2_ reduction performance was systematically evaluated under visible-light irradiation. Positron annihilation lifetime spectroscopy and spherical aberration-corrected scanning transmission electron microscopy confirmed the existence of oxygen vacancies and the successful construction of heterointerfaces. The optimized 1.0FM/V_O_-BWO heterojunction exhibited 11.8-fold and 1.5-fold enhancements in photocatalytic activity for CO and CH_4_ production compared to pristine Bi_2_WO_6_, respectively. During the simulated catalytic reduction process, CO_2_ molecules demonstrated enhanced adsorption and favorable activation on the 1.0FM/V_O_-BWO surface, thereby boosting photocatalytic activity. In situ molecular spectroscopy was employed to detect molecular coordination structures and key intermediates on the catalyst surface, elucidating the photocatalytic CO_2_ reduction pathway. Ultraviolet photoelectron spectroscopy further validated the authenticity of charge transfer pathways and their efficiency in the FM/V_O_-BWO system. Based on these mechanistic insights, we propose a defect-mediated Z-scheme charge transfer mechanism that synergistically enhances charge separation efficiency and surface catalysis to elevate photocatalytic CO_2_ reduction performance.

## 2. Results and Discussion

**Scheme 1:**
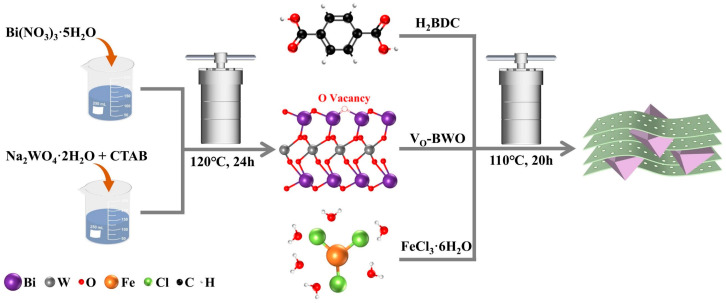
The schematic synthetic route of FM/V_O_-BWO.

**Figure 1:**
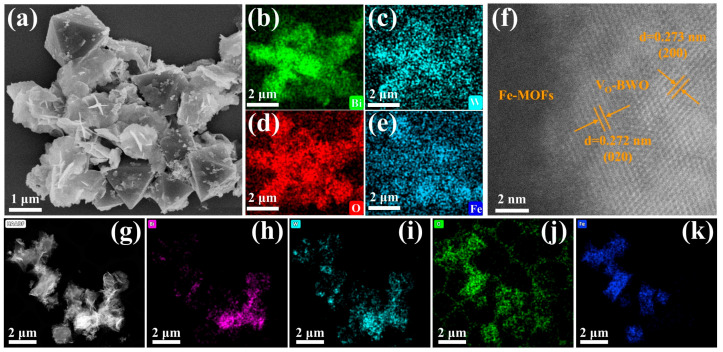
(**a**) SEM images and (**b**–**e**) EDS elemental maps of 1.0FM/V_O_-BWO. (**f**) AC-STEM images of 1.0FM/V_O_-BWO. (**g**) HAADF image and EDS elemental maps of (**h**) Bi, (**i**) W, (**j**) O, and (**k**) Fe elements in 1.0FM/V_O_-BWO.

The fabrication process of the FM/V_O_-BWO heterojunction catalyst is illustrated in Scheme 1. Initially, ultrathin V_O_-BWO nanosheets enriched with oxygen vacancies were synthesized via a CTAB-assisted hydrothermal method using Bi(NO_3_)_3_·5H_2_O and Na_2_WO_4_·2H_2_O as precursors. Subsequently, the as-prepared V_O_-BWO was dispersed into a mixed solution containing FeCl_3_·6H_2_O and H_2_BDC (molar ratio 2:1), followed by a solvothermal reaction to obtain FM/V_O_-BWO hybrids with varying mass ratios. The crystalline phases and morphologies were characterized by scanning electron microscopy (SEM) and spherical aberration-corrected scanning transmission electron microscopy (AC-STEM). As shown in Figure S1, V_O_-BWO exhibits a cross-stacked ultrathin nanosheet morphology, while Fe-MOFs display uniform octahedral structures. SEM images of the FM/V_O_-BWO hybrids (Figure 1a) demonstrate the homogeneous attachment of V_O_-BWO nanosheets to Fe-MOF octahedra. Elemental mapping analysis further confirms the spatial distribution of components: Bi, W, and O are uniformly dispersed across the nanosheets (Figure 1b–e,g–k), whereas Fe is exclusively localized on the encapsulated octahedra. Table S1 presents the relative content of elements in the hybrid material. The observed elemental ratios align well with the theoretical stoichiometry of the designed composite, further confirming the successful integration of the two materials during synthesis. High-resolution AC-STEM imaging (Figure 1f) reveals distinct interfacial characteristics between the two phases. V_O_-BWO displays clear lattice fringes with interplanar spacings of 0.272 nm and 0.273 nm, corresponding to the (020) and (200) crystallographic planes, respectively [28]. In contrast, Fe-MOFs exhibit amorphous-like features without observable lattice fringes, confirming the successful construction of heterojunction interfaces. X-ray diffraction (XRD) patterns were employed to investigate the crystallinity. As depicted in Figure S2, the diffraction peaks of Fe-MOFs match the literature-reported data [29]. Both BWO and V_O_-BWO samples exhibit characteristic peaks indexed to orthorhombic Bi_2_WO_6_ (JCPDS No. 73-2020) [30]. Notably, pristine BWO displays sharp diffraction peaks indicative of high crystallinity, while V_O_-BWO shows attenuated peak intensities due to oxygen vacancy-induced lattice distortion. The XRD pattern of 1.0FM/V_O_-BWO closely resembles that of V_O_-BWO, with no discernible Fe-MOFs signals, likely attributable to their low mass fraction. Nevertheless, the slight reduction in diffraction peak intensity upon Fe-MOFs incorporation provides indirect evidence for successful heterojunction formation.

Raman spectroscopy was employed to probe the molecular structural evolution of the synthesized materials. As depicted in Figure S3, the Fe-MOFs spectrum displays four characteristic vibrational modes: Peaks at 1611.8 and 1435.9 cm^−1^ are assignable to in-plane C=C stretching and ring breathing modes of the benzene carbon framework, respectively; the 1143.7 cm^−1^ feature corresponds to in-plane C-H bending vibrations, while the 862.9 cm^−1^ peak arises from out-of-plane C-H wagging modes. For pristine BWO, dominant Raman bands at 153.2 and 306.6 cm^−1^ are attributed to Bi-O bending and W-O stretching vibrations within WO_6_ octahedra. The 713.8 cm^−1^ mode corresponds to the distortion in W−O interaction, whereas peaks at 794.1 and 825.6 cm^−1^ originate from O-W-O symmetric stretching vibrations [31,32]. Notably, the V_O_-BWO sample exhibits attenuated Raman peak intensities compared to pristine BWO, accompanied by a redshift of the 795.3 cm^−1^ band and a blueshift of the 821.3 cm^−1^ feature. Bi-O and W-O bending vibrations cause these spectral changes. In the 1.0FM/V_O_-BWO composite, the co-existence of Vo-BWO and Fe-MOFs is evidenced by the simultaneous presence of their characteristic Raman signatures. Remarkably, the composite demonstrates significant peak intensity reduction relative to individual components, indicative of interfacial electronic interactions between V_O_-BWO and Fe-MOFs.

**Figure 2:**
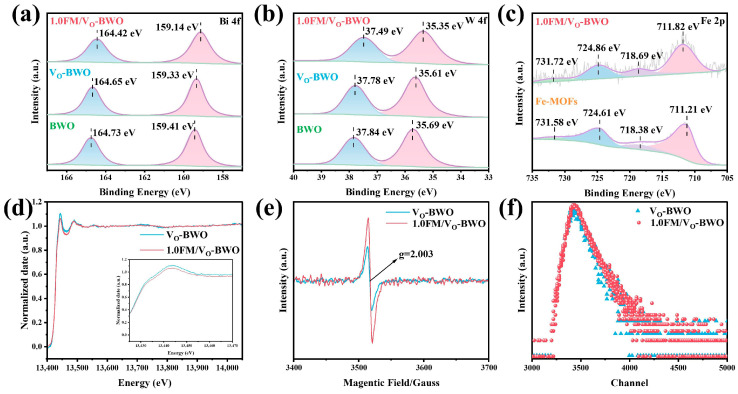
High-resolution XPS spectra of the core levels of Bi 4f (**a**) and W 4f (**b**) on BWO, V_O_-BWO, and 1.0FM/V_O_-BWO, and Fe 2p (**c**) on Fe-MOFs and 1.0FM/V_O_-BWO. (**d**) XANES Bi L_3_ edge spectra of V_O_-BWO and 1.0FM/V_O_-BWO. (**e**) EPR spectra of V_O_-BWO and 1.0FM/V_O_-BWO. (**f**) Positron annihilation spectra of V_O_-BWO and 1.0FM/V_O_-BWO.

X-ray photoelectron spectroscopy (XPS) was conducted to elucidate the surface elemental composition and chemical states of the synthesized materials. For pristine BWO, the deconvoluted peaks at 164.73 eV (Bi 4f_5/2_) and 159.41 eV (Bi 4f_7/2_) correspond to Bi^3+^ in the Bi-O-W lattice [33]. The high-resolution W4f spectrum has two peaks at 37.83 eV and 35.69 eV, assigned to the W 4f_5/2_ and W 4f_7/2_ orbitals [34]. Notably, V_O_-BWO exhibits 0.2–0.3 eV negative shifts in Bi 4f and W 4f binding energies compared to pristine BWO, indicative of electron cloud densification around metal centers induced by oxygen vacancy-mediated charge redistribution [35]. This trend is further amplified in 1.0FM/V_O_-BWO, where additional negative shifts (Δ = 0.1–0.2 eV relative to V_O_-BWO) suggest that there is electron transfer between Fe-MOFs and V_O_-BWO (Figure 2a,b). Two typical XPS peaks at 530.29 eV and 531.84 eV in V_O_-BWO can be attributed to lattice oxygen and oxygen defects, respectively [36]. Concurrently, a new O 1s component appeared at 1.0FM/V_O_-BWO at 533.44 eV, due to the formation of C-O bonds at the heterojunction interface (Figure S4) [37]. Intriguingly, the Fe 2p spectrum of 1.0FM/V_O_-BWO displays positive binding energy shifts of 0.25 eV (Fe 2p_1/2_: 724.86 eV) and 0.61 eV (Fe 2p_3/2_: 711.82 eV) compared to pristine Fe-MOFs (724.61 eV and 711.21 eV, respectively) (Figure 2c) [38]. The results of XPS confirm the successful construction of heterojunction. To further validate the surface chemical states, X-ray absorption fine structure (XAFS) spectroscopy was systematically employed. As illustrated in Figure 2d, the Bi L-edge XANES spectra of V_O_-BWO and 1.0FM/V_O_-BWO exhibit analogous near-edge features. Notably, compared to V_O_-BWO, the absorption edge of 1.0FM/V_O_-BWO demonstrates a distinct leftward shift accompanied by attenuated XANES intensity. This observation indicates a reduced oxidation state of Bi (denoted as Bi^δ+^, 0 < δ < 3) in 1.0FM/V_O_-BWO relative to the Bi^3+^ species in pristine V_O_-BWO, consistent with the XPS-derived conclusions [35].

The oxygen vacancy concentration was quantitatively analyzed by electron paramagnetic resonance (EPR) spectroscopy. As shown in Figure 2e, V_O_-BWO exhibits a distinct paramagnetic signal at g = 2.003, characteristic of unpaired electrons localized at oxygen vacancy sites. The enhanced EPR signal intensity observed in 1.0FM/V_O_-BWO (compared to V_O_-BWO) suggests increased oxygen vacancy density, likely mediated by electron transfer processes at the Fe-MOFs/V_O_-BWO interface. Positron annihilation lifetime spectroscopy (PALS), a well-established technique for probing localized microstructural defects, was utilized to comparatively analyze defect structures and concentrations in V_O_-BWO and 1.0FM/V_O_-BWO. As shown in Figure 2f and Table S2, the acquired PALS decay spectra for all samples exhibit three distinct lifetime components. The shortest component τ_1_ corresponds to positron annihilation at oxygen vacancy clusters, while the intermediate component τ_2_ arises from positrons trapped at isolated oxygen vacancies. The longest component τ_3_ is attributed to positronium formation in large holes [19,39]. It is worth noting that the τ_1_ and τ_2_ values of 1.0FM/V_O_-BWO (221.3 ps and 320.3 ps, respectively) are improved compared to those of V_O_-BWO (τ_1_ = 216.3 ps, τ_2_ = 317.3 ps). The relative intensity (I_2_) of the oxygen vacancy-associated τ_2_ component, proportional to defect density, increases from 52.3% for V_O_-BWO to 57.3% for 1.0FM/V_O_-BWO. These PALS metrics demonstrate that heterojunction engineering effectively amplifies oxygen vacancy concentrations while maintaining structural integrity.

**Figure 3:**
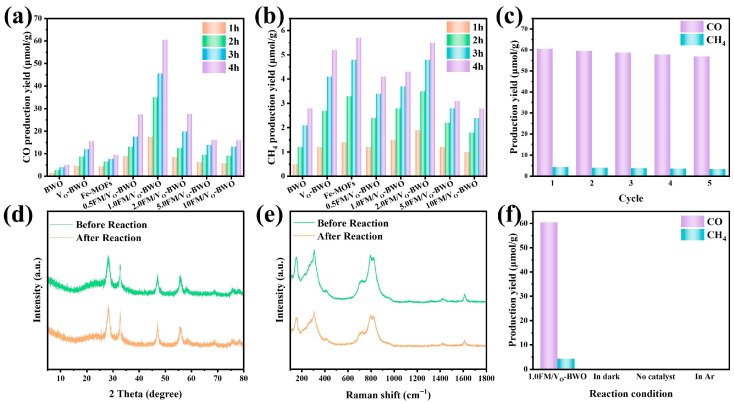
Photocatalytic activities of CO_2_ reduction over BWO, V_O_-BWO, Fe-MOFs, and FM/V_O_-BWO samples at different proportions during 4 h experiments performed under visible-light irradiation: time course of (**a**) CO and (**b**) CH_4_ production yields. (**c**) Cycling measurements of 1.0FM/V_O_-BWO toward CO_2_ photoreduction. (**d**,**e**) XRD and Raman comparison before and after reaction for the 1.0FM/V_O_-BWO sample. (**f**) Control experiments under different conditions of 1.0FM/V_O_-BWO.

The optical absorption characteristics of the synthesized materials were systematically investigated via UV–Vis diffuse reflectance spectroscopy (DRS). All samples exhibited broad visible-light absorption edges spanning 400–800 nm (Figure S5a), prompting subsequent photocatalytic CO_2_ reduction evaluations under visible-light irradiation (300 W Xe lamp with a 420 nm cutoff filter). After 4 h of continuous irradiation, gas chromatographic analysis revealed CO as the dominant reduction product, accompanied by minor CH_4_ generation (Figure 3a,b). Notably, pristine BWO demonstrated limited catalytic activity, yielding 5.1 μmol/g CO and 2.8 μmol/g CH_4_, while Fe-MOFs alone showed modest improvements (9.5 μmol/g CO, 5.7 μmol/g CH_4_). Oxygen vacancy engineering in V_O_-BWO enhanced CO and CH_4_ yields by 3.0- and 1.8-fold relative to BWO, respectively, attributable to defect-mediated electron trapping that prolongs charge carrier lifetimes. The performance is further optimized by introducing Fe-MOF into V_O_-BWO to form a heterojunction. The 1.0FM/V_O_-BWO composite showed optimal photocatalytic performance, achieving CO and CH_4_ production rates of 60.48 and 4.3 μmol/g, respectively, after 4 h of light irradiation, corresponding to 11.8-fold and 1.5-fold enhancements compared to pristine BWO. This performance surpasses previously reported BWO- and Fe-MOF-based photocatalysts (Table S3). However, excessive Fe-MOF loading (>1 wt%) progressively diminished catalytic efficiency, underscoring the necessity of optimal interfacial coupling between Fe-MOFs and V_O_-BWO for efficient charge transfer dynamics. This non-monotonic dependency highlights the critical balance between defect engineering and heterojunction design in photocatalytic systems.

The 1.0FM/V_O_-BWO composite demonstrated negligible attenuation in CO and CH_4_ production yields after five consecutive photocatalytic cycles (Figure 3c), exhibiting cyclic stability under prolonged irradiation. To evaluate structural stability, post-reaction characterization was performed via XRD, Raman, and SEM. The XRD pattern and Raman spectroscopy showed that the crystal phase of the catalyst was similar before and after the reaction (Figure 3d,e). In addition, the SEM images showed that the morphological characteristics of both the post-reaction sample and the sample subjected to five consecutive cycles remain well-preserved (Figure S6). These comprehensive analyses verify the exceptional operational stability of 1.0FM/V_O_-BWO under reactive conditions. A control test was performed to verify the carbon source of the photocatalytic CO_2_ reduction products. As shown in Figure 3f, no product was detected in the absence of CO_2_, indicating that both CO and CH_4_ originated from the reactant CO_2_. Also, no products were detected in the absence of light and a catalyst. These experiments demonstrated that CO and CH_4_ were only produced in the presence of a light source, CO_2_, and a catalyst simultaneously.

**Figure 4:**
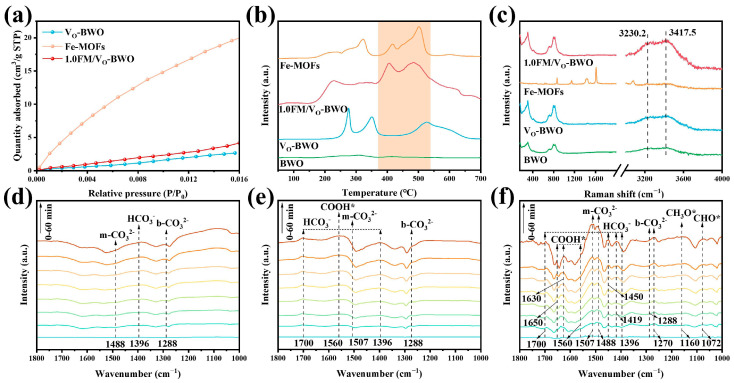
(**a**) CO_2_ adsorption isotherms of V_O_-BWO, Fe-MOFs, and 1.0FM/V_O_-BWO. (**b**) CO_2_-TPD of BWO, V_O_-BWO, Fe-MOFs, and 1.0FM/V_O_-BWO. (**c**) In situ Raman spectra of BWO, V_O_-BWO, Fe-MOFs, and 1.0FM/V_O_-BWO with adsorbed H_2_O. In situ DRIFTS spectra of BWO (**d**), V_O_-BWO (**e**), and 1.0FM/V_O_-BWO (**f**) in photocatalytic CO_2_ reduction.

To elucidate the mechanism of the CO_2_ photoreduction process over the 1.0 FM/V_O_-BWO heterojunction catalyst, we have explored both its surface catalytic chemistry and charge dynamics. The adsorption and activation between the catalyst and the reactant CO_2_ molecules were first explored through characterizations. It can be seen from Figure S7 and Table S4 that pure BWO has a small specific surface area of 29.549 m^2^/g. After the oxygen vacancy is introduced, the particular surface area of V_O_-BWO is slightly increased. The specific surface area of Fe-MOFs is large, reaching 598.073 m^2^/g. The specific surface area of the 1.0FM/V_O_-BWO sample was effectively improved by introducing Fe-MOFs, which was three times that of pure BWO. The adsorption capacity of the catalyst for CO_2_ is also an essential factor affecting photocatalytic efficiency. In Figure 4a, Fe-MOFs exhibited higher CO_2_ capture capacity than V_O_-BWO. Its larger specific surface area and abundant pore structure enhanced the CO_2_ adsorption capacity of the 1.0 FM/V_O_-BWO sample. CO_2_-TPD also obtained further understanding of the interaction between the catalyst and the CO_2_ molecule. As shown in Figure 4b, no obvious CO_2_ desorption peaks were observed for pure BWO, two CO_2_ desorption peaks appeared for V_O_-BWO at 276 °C and 350 °C, whereas the desorption peaks for Fe-MOFs appeared at higher temperatures (417 °C and 500 °C). At 350–550 °C, the 1.0FM/V_O_-BWO sample showed strong chemisorption of CO_2_, indicating that the 1.0FM/V_O_-BWO sample has the best CO_2_ chemisorption capacity, which is conducive to the photocatalytic conversion of CO_2_. To further investigate the acidic sites in the 1.0FM/V_O_-BWO hybrid material, NH_3_-TPD experiments were conducted. Generally, signals observed in the low-temperature region (50–200 °C) are attributed to weak acidic sites, while ammonia desorption signals in the range of 200–400 °C correspond to medium-strength acidic sites. Strong acidic sites typically exhibit desorption signals at temperatures exceeding 400 °C. As shown in Figure S8, the NH_3_-TPD profile of 1.0FM/V_O_-BWO displays two consecutive desorption peaks at 86 and 111 °C assigned to weak acid sites, a distinct desorption peak at 242 °C corresponding to medium-strength acid sites, and two prominent desorption peaks (406 and 489 °C) observed in the strong acidic region. These results indicate that the unique structure of 1.0FM/V_O_-BWO provides a more open structural environment for exposing acidic sites, which facilitates the more efficient transportation of reactant molecules to the active acidic centers, thereby contributing to its enhanced catalytic activity [40].

Considering H_2_O is a relatively important hydrogen source, exploring the adsorption and coordination structure between H_2_O molecules and catalysts is also a necessary condition to reveal the reaction mechanism. The water contact angle test was used to detect the absorption of H_2_O molecules on the sample surface. All the samples in Figure S9 have contact angles less than 90° and are hydrophilic surfaces. The contact angles of V_O_-BWO and Fe-MOFs were 45.1°and 59.7°, respectively. After modification with Fe-MOFs, the contact angle of 1.0FM/V_O_-BWO was reduced to 36.6°. It is shown that the H_2_O molecules are better wetted on the 1.0 FM/V_O_-BWO surface, which is more favorable for the adsorption of H_2_O molecules. Subsequently, in situ Raman spectroscopy was used to study the coordination structure between the catalyst and the H_2_O molecule. Figure 4c shows that when H_2_O molecules are adsorbed on the catalyst surface, two O-H stretching vibrational peaks are displayed in the Raman spectrum of the sample. The characteristic peak located at 3230.2 cm^−1^ is associated with four-coordinated hydrogen-bonded water (4-HB·H_2_O), and the characteristic peak located at 3417.5 cm^−1^ is attributed to two-coordinated hydrogen-bonded water (2-HB·H_2_O) [41]. The 1.0FM/V_O_-BWO sample has the highest intensity of the characteristic peaks, which indicates that the 1.0FM/V_O_-BWO heterojunction catalyst surface is more favorable for the adsorption, activation, and coordination of H_2_O molecules.

In situ diffuse reflectance Fourier transform infrared spectroscopy (In Situ DRIFTS) experiments were carried out to better understand the intermediates and transformation pathways of the photocatalytic CO_2_ reduction reaction process. Figure 4d–f and Figure S10 show the changes in multiple carbon-active intermediates adsorbed on the surface of the catalyst under light conditions from 0 to 60 min. The peaks at 1072 and 1160 cm^−1^ are attributed to CHO* and CH_3_O*, respectively [42], which are intermediates in the reduction of CO_2_ to CH_4_. The peaks at 1270 and 1288 cm^−1^ belong to bidentate carbonates (b-CO_3_^2−^), and the peaks at 1488 and 1507 cm^−1^ belong to monodentate carbonates (m-CO_3_^2−^) [35,43]. The peaks at 1396, 1419, 1450, and 1700 cm^−1^ originate from the symmetric and asymmetric stretching of bicarbonate (HCO_3_^−^) [44]. The presence of COOH* at 1560, 1630, and 1650 cm^−1^ is considered a key intermediate in reducing CO_2_ to CO [45]. And the intensity of these observed intermediate peaks increased with increasing light duration. In contrast, the peak intensities of the intermediates in the IR spectra of BWO, V_O_-BWO, and Fe-MOFs are much lower, suggesting that they are less capable of CO_2_ conversion under light than 1.0 FM/V_O_-BWO. Experimental results from In Situ DRIFTS confirm that the formation of key intermediates enhances the activity of photocatalytic CO_2_ reduction.

**Figure 5:**
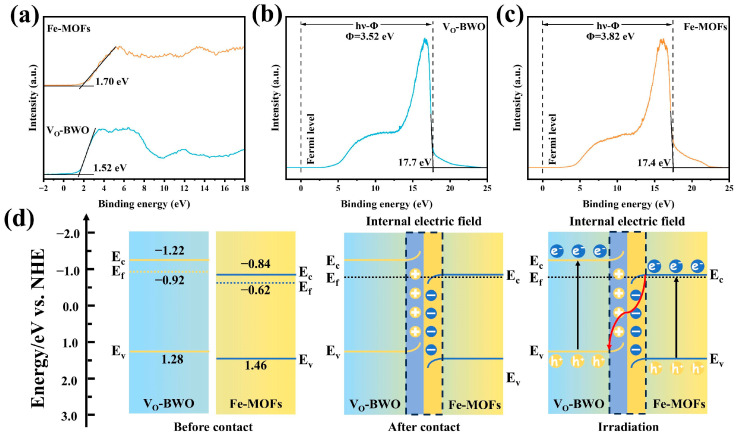
(**a**) XPS valence band spectra of V_O_-BWO and Fe-MOFs. (**b**,**c**) UPS spectra of V_O_-BWO and Fe-MOFs. (**d**) Mechanism of photogenerated carriers of Z-scheme heterojunction.

The light-induced interfacial charge transfer process is an important way to investigate the mechanism of the CO_2_ photoreduction reaction. Tauc plots, XPS valence band spectroscopy (XPS-VB), and ultraviolet photoelectron spectroscopy (UPS) were first utilized to understand the energy band structures of V_O_-BWO and Fe-MOFs to investigate the carrier migration direction at the Fe-MOFs/V_O_-BWO heterojunction interface. As shown in Figure S5b, the bandgap value of Fe-MOFs is 2.3 eV, and introducing oxygen vacancies reduces the bandgap value of V_O_-BWO from 2.6 eV to 2.5 eV for BWO. The valence band values of V_O_-BWO and Fe-MOFs were determined using XPS-VB and were calculated to be 1.28 eV and 1.46 eV concerning the standard hydrogen electrode, respectively (Figure 5a). The calculation formula is E_VB-NHE_ = φ + E_VB-XPS_ − 4.44. E_VB-NHE_ is the value of the valence band for the standard hydrogen electrode, φ is the power function of the instrument (4.2 eV), and E_VB-XPS_ is the value of the valence band measured by XPS-VB [46]. The conduction band values of V_O_-BWO and Fe-MOFs to the standard hydrogen electrode can be calculated as −1.22 eV and −0.84 eV, respectively, by the formula E_g_ = E_VB_ − E_CB_, where E_g_ is the bandgap value. Then, the work function of V_O_-BWO and Fe-MOFs is calculated by using UPS (Figure 5b,c) as 3.52 eV and 3.82 eV, respectively, and the formula is Φ = hv − E_cutoff_, where Φ is the work function, hv is the energy of the excited photon (21.22 eV), and E_cutoff_ is the binding energy of the secondary electron to the edge. Since the work function (Φ) is equal to the difference between the vacuum energy level (E_vac_) and the Fermi energy level (E_f_), Φ = E_vac_ − E_f_. Thus, the Fermi energy levels of V_O_-BWO and Fe-MOFs to the standard hydrogen electrode can be obtained as −0.92 eV and −0.62 eV, respectively [47]. When the two are in close contact, the electrons in V_O_-BWO are spontaneously transferred to Fe-MOFs until E_f_ equilibrium. The electrons will accumulate at the Fe-MOFs interface and decrease at the V_O_-BWO interface, resulting in a downward bending of the energy bands of Fe-MOFs and an upward bending of the energy bands of V_O_-BWO, creating an internal electric field from V_O_-BWO to Fe-MOFs at the heterojunction interface. Under light irradiation, the built-in electric field drives the photogenerated electrons to migrate from the conduction band of Fe-MOFs to the valence band of V_O_-BWO, which achieves the effective separation of photogenerated carriers (Figure 5d). Consequently, the charge transfer between V_O_-BWO and Fe-MOFs belongs to the Z-scheme heterojunction pathway.

**Figure 6:**
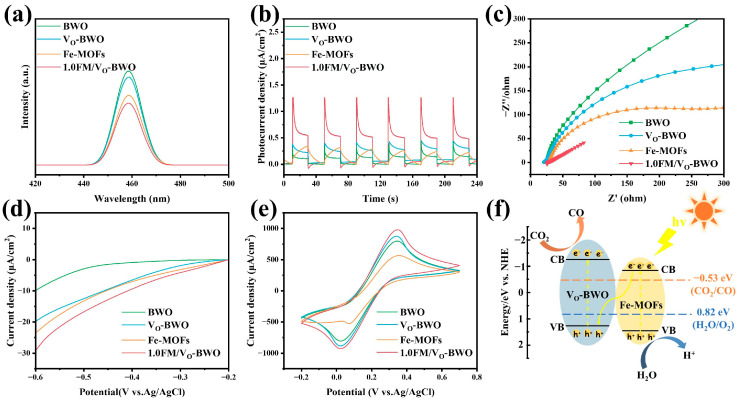
(**a**) PL spectra, (**b**) transient photocurrent spectra, (**c**) electrochemical impedance spectra, (**d**) LSV, and (**e**) CV of BWO, V_O_-BWO, Fe-MOFs, and 1.0FM/V_O_-BWO. (**f**) The proposed mechanism of CO_2_ photoreduction is attributed to the formation of a Z-scheme heterojunction within FM/V_O_-BWO nanocomposites.

The separation dynamics and transportation efficiency of photogenerated electron–hole pairs were systematically investigated through photoluminescence (PL) spectroscopy coupled with comprehensive photoelectrochemical characterization. As shown in Figure 6a, the photoluminescence intensity of the 1.0FM/V_O_-BWO sample was the weakest at 459 nm, demonstrating superior suppression of charge carrier recombination through the established direct Z-scheme charge transfer pathway. Complementary to this, transient photocurrent responses under intermittent irradiation revealed that 1.0FM/V_O_-BWO generated the highest and most stable photocurrent density (Figure 6b), confirming enhanced charge separation kinetics. The photocurrent response characteristics of the material exhibit consistency with those of n-type semiconductor materials, which aligns with the findings reported in the literature [48,49,50,51,52]. Electrochemical impedance spectroscopy (EIS) Nyquist plots (Figure 6c) further corroborated these findings, where 1.0FM/V_O_-BWO displayed the smallest Nyquist arc radius, corresponding to the lowest charge transfer resistance and most efficient interfacial charge migration [53,54,55,56]. Linear sweep voltammetry (LSV) analysis demonstrated that 1.0FM/V_O_-BWO required the lowest overpotential for hydrogen evolution (Figure 6d), indicating optimized proton reduction kinetics that synergistically enhance CO_2_ reduction through hydrogen-assisted pathways. In the cyclic voltammetry (CV) curves (Figure 6e), the reduction peaks are near the low potentials and the oxidation peaks are near the high potentials. In addition, it can be observed that the redox peak area of 1.0FM/V_O_-BWO is the largest, reflecting its excellent charge storage capacity and surface redox activity. The regularity of the photoelectric chemical test results is consistent with that reported in the literature [57]. Collectively, these multiscale analyses substantiate that the synergistic integration of oxygen vacancies and Z-scheme heterojunction architecture synergistically suppresses charge carrier recombination while preserving strong redox potentials, thereby significantly enhancing photocatalytic CO_2_ conversion efficiency. The vacancy-mediated defect states and interfacial electric field jointly facilitate directional electron transfer from Fe-MOFs to V_O_-BWO, aligning with previously proposed mechanisms.

Based on the aforementioned findings, we propose a Z-scheme heterojunction mechanism governing the photocatalytic CO_2_ reduction over the FM/V_O_-BWO nanocomposite. Upon aqueous contact, H_2_O molecules adsorb onto the 1.0FM/V_O_-BWO surface through a four-coordinated hydrogen bond and a two-coordinated hydrogen bond, achieving adsorption equilibrium, while CO_2_ is preferentially immobilized at oxygen vacancy-enriched sites via C-O interactions facilitated by abundant surface-active centers. Under simulated solar irradiation, photoexcitation induces electron-hole pair generation in Fe-MOFs and V_O_-BWO at their respective conduction (CB) and valence bands (VB). The interfacial charge dynamics proceed through rapid electron migration from the CB of Fe-MOFs to the VB of V_O_-BWO, where recombination with photogenerated holes effectively suppresses bulk charge loss. On the photocatalyst surface, the photogenerated holes (h^+^) retained in the valence band (VB) of Fe-MOFs oxidize water molecules to generate H^+^, establishing a proton source for CO_2_ reduction. During water oxidation, the photogenerated holes drive proton (H^+^) migration from the bulk solution to reduction sites through electrostatic attraction. At the CO_2_ reduction sites, these protons (H^+^) synergistically interact with photogenerated electrons (e^−^) accumulated in the conduction band (CB) of V_O_-BWO to participate in multi-step reduction reactions, as demonstrated by CO_2_ + 2H^+^ + 2e^−^ → CO + H_2_O and CO_2_ + 8H^+^ + 8e^−^ → CH_4_ + 2H_2_O (Figure 6f) [37]. This Z-scheme configuration synergistically preserves the strong redox potentials of individual components while enhancing charge separation efficiency, with oxygen vacancies in V_O_-BWO serving dual roles as CO_2_ adsorption sites and electron reservoirs to sustain catalytic activity.

This study addresses the urgent need for developing efficient photocatalytic systems to convert CO_2_ into renewable hydrocarbon fuels, a critical pathway toward sustainable energy conversion and carbon neutrality. The rational integration of oxygen vacancy engineering and Z-scheme heterojunction construction overcomes two fundamental limitations in conventional photocatalysts: insufficient visible-light utilization and the rapid recombination of photogenerated carriers. By systematically elucidating the dual role of oxygen vacancies in promoting CO_2_ adsorption/activation and regulating charge dynamics, this work provides fundamental insights into defect-mediated photocatalytic mechanisms. The remarkable activity enhancement demonstrates the feasibility of coupling metal–organic frameworks with vacancy-rich semiconductors for multi-functional catalytic systems. Furthermore, the in situ spectroscopic characterization methodology establishes a robust framework for tracking surface reaction pathways, offering valuable guidance for designing next-generation photocatalysts. These findings not only advance the mechanistic understanding of interfacial charge transfer in complex heterostructures but also present a scalable strategy to mitigate greenhouse gas emissions through solar-driven fuel production.

## 3. Conclusions

This study successfully demonstrates the rational integration of oxygen vacancy engineering and Z-scheme heterojunction architecture to achieve efficient photocatalytic CO_2_ reduction. The 1.0FM/V_O_-BWO composite exhibited excellent CO production (60.48 μmol/g) and stability over five cycles, exceeding previously reported Bi_2_WO_6_- and Fe-MOF-based systems. By constructing Fe-MOF/V_O_-BWO composites, we synergistically addressed critical limitations in charge recombination and CO_2_ activation. The CO_2_-TPD and CO_2_ adsorption curves verify that the oxygen vacancy in V_O_-BWO creates an electron-rich site for CO_2_ chemisorption. In Situ DRIFTS and Raman spectroscopy elucidated critical intermediates (e.g., COOH*, CHO*) and four-coordinated hydrogen-bonded water and two-coordinated hydrogen-bonded water, underscoring the role of surface chemistry in reaction kinetics. The Z-scheme charge transfer pathway, driven by interfacial electric fields, enables the efficient separation of photogenerated carriers while maintaining strong redox potentials, as demonstrated by UPS and photoelectrochemical analyses. These insights highlight the dual functionality of defects and heterojunctions in photocatalyst design. Future work will focus on scaling reactor configurations and exploring industrial applicability, paving the way for scalable solar-driven carbon neutrality solutions.

## 4. Experimental Section

### 4.1. Materials

Bismuth nitrate pentahydrate (Bi(NO_3_)_3_·5H_2_O), sodium tungstate dihydrate (Na_2_WO_4_·2H_2_O), cetyltrimethylammonium bromide (CTAB), iron chloride hexahydrate (FeCl_3_·6H_2_O), 1,4-benzenedicarboxylic acid (H_2_BDC), N,N-dimethylformamide (DMF), acetonitrile (CH_3_CN) and barium fluoride (BaF_2_) were purchased from Aladdin Reagent Co. (Shanghai, China). Sodium sulfate (Na_2_SO_4_), potassium hexacyanoferrate (K_3_[Fe(CN)_6_]), potassium ferrocyanide (K_4_[Fe(CN)_6_]) were obtained from Sinopharm Chemical Reagent Co. (Beijing, China). Ethanol (C_2_H_6_O) was sourced from Yunnan Jingrui Technology Co., (Yuxi, China). All reagents in the experiments were of analytical grade without any disposal. And the water used in the experiment was deionized water (DI).

### 4.2. Synthesis of Layered Bi_2_WO_6_

First, 2 mmol of Bi(NO_3_)_3_·5H_2_O and 1 mmol of Na_2_WO_4_·2H_2_O were dissolved in 60 mL of deionized water under continuous stirring for 12 h at room temperature. The homogeneous mixture was then transferred to a 100 mL Teflon-lined stainless-steel autoclave and hydrothermally treated at 120 °C for 24 h. After natural cooling to room temperature, the precipitate was collected via centrifugation and repeatedly washed with deionized water and ethanol to eliminate surface impurities. Finally, the product was obtained by drying at 80 °C for 12 h.

### 4.3. Synthesis of V_O_-Bi_2_WO_6_ Nanosheets

Initially, 2 mmol of Bi(NO_3_)_3_·5H_2_O was dispersed in 30 mL of deionized water. Separately, 1 mmol of Na_2_WO_4_·2H_2_O and 0.05 g of cetyltrimethylammonium bromide (CTAB) were dissolved in another 30 mL of deionized water. The Bi(NO_3_)_3_·5H_2_O solution was gradually added to the Na_2_WO_4_·2H_2_O/CTAB mixture under vigorous stirring for 12 h at room temperature. The resultant solution was subjected to hydrothermal reaction in a 100 mL Teflon-lined autoclave at 120 °C for 24 h. After cooling to ambient temperature, the product was isolated by centrifugation, washed with deionized water and ethanol, and dried at 80 °C for 12 h to yield Bi_2_WO_6_ nanosheets with surface oxygen vacancies (denoted as V_O_-BWO).

### 4.4. Synthesis of Fe-MOFs

A precursor solution was prepared by dispersing 2.48 mmol of FeCl_3_·6H_2_O and 1.24 mmol of 1,4-benzenedicarboxylic acid (H_2_BDC) in 20 mL of N,N-dimethylformamide (DMF) under 1 h of magnetic stirring at room temperature. The mixture was then sealed in a 25 mL Teflon-lined autoclave and heated at 110 °C for 20 h. After natural cooling, the brown precipitate was centrifuged, washed three times with N,N-dimethylformamide (DMF), and dried overnight at 80 °C to obtain Fe-based metal–organic frameworks (Fe-MOFs).

### 4.5. Synthesis of Fe-MOFs/V_O_-Bi_2_WO_6_ Composites

V_O_-Bi_2_WO_6_ powder (denoted as V_O_-BWO) was uniformly dispersed in 50 mL of DMF via 1 h of ultrasonication. Subsequently, FeCl_3_·6H_2_O and H_2_BDC were added to the suspension at a molar ratio of 2:1, followed by 1 h of stirring at room temperature. The reaction system was transferred to a 100 mL Teflon-lined autoclave and maintained at 110 °C for 20 h. The cooled product was centrifuged, rinsed with DMF, and dried overnight at 80 °C. Composites with Fe-MOFs to V_O_-BWO mass ratios of 0.5%, 1%, 2%, 5%, and 10% were synthesized and labeled as 0.5FM/V_O_-BWO, 1.0FM/V_O_-BWO, 2.0FM/V_O_-BWO, 5.0FM/V_O_-BWO, and 10FM/V_O_-BWO, respectively.

### 4.6. General Characterization

Powder X-ray diffraction (PXRD) patterns were acquired by a Bruker D8 Advance (Bruker, Bremen, Germany) instrument with a Cu Kα source (λ = 0.1541 nm). Morphological and crystallographic information was acquired using scanning electron microscopy (SEM, Zeiss Gemini 500, Carl Zeiss AG, Baden-Württem, Germany) and scanning transmission electron microscopy (STEM, Thermofisher Spectra 300, Thermo Fisher Scientific, Waltham, MA, USA). The samples’ specific surface area and pore structure were characterized using the Quantachrome NOVA4200e (Anton Paar, Boynton Beach, FL, USA) instrument. Raman spectroscopy was conducted utilizing a Renishaw inVia spectrometer (Renishaw, London, UK) with an excitation wavelength of 532 nm, covering a wavenumber range from 100 to 1800 cm^−1^. X-ray photoelectron spectroscopy (XPS) and valence-band X-ray photoelectron spectroscopy (VB-XPS) measurements were performed using a Thermo escalab 250Xi spectrometer (Thermo Fisher Scientific, Waltham, MA, USA) with Al Kα X-ray radiation. Electron paramagnetic resonance (EPR) measurements were performed at 25 °C on an EPR spectrometer (Bruker A300–10/12, Bruker, Bremen, Germany). The light absorption properties and bandgap of the samples were characterized using UV–Vis diffuse reflection spectrophotometry (UV-DRS, PerkinElmer LAMBDA 1050+, PerkinElmer, Waltham, MA, USA). The water contact angle measurements were recorded using a KRUSS DSA 100 (KRUSS, Hamburg, Germany) contact angle meter. The CO_2_ adsorption isotherms were obtained at 25 °C using a high-performance specific surface area and aperture analyzer (JW-BK300, Beijing Jingwei Gao Bo Science and Technology Co., LTD, Beijing, China). The CO_2_ temperature programmed desorption (CO_2_-TPD) was conducted over a temperature range of 50 to 700 °C using a fully automated chemisorption instrument (BDS-Chem C200, BSD Instrument, Beijing, China). The NH_3_ temperature programmed desorption (NH_3_-TPD) was conducted over a temperature range of 50 to 600 °C using a fully automated chemisorption instrument (BDS-Chem C200, BSD Instrument, Beijing, China). UPS measurements were conducted using a monochromatic He I light source (21.22 eV) with an applied bias of 10.0 V, utilizing a Thermofisher ESCALAB 250Xi analyzer (Thermo Fisher Scientific, Waltham, MA, USA). Photoluminescence (PL) spectra were recorded using an F-4600 HITACHI spectrofluorometer (Hitachi, Tokyo, Japan).

### 4.7. In Situ Raman Spectroscopy

A precursor solution was prepared by dispersing 2.48 mmol of FeCl_3_·6H_2_O and 1.24 mmol of 1,4-benzenedicarboxylic acid (H_2_BDC) in 20 mL of N,N-dimethylformamide (DMF) under 1 h of magnetic stirring at room temperature. The mixture was then sealed in a 25 mL Teflon-lined autoclave and heated at 110 °C for 20 h. After natural cooling, the brown precipitate was centrifuged, washed three times with DMF, and dried overnight at 80 °C to obtain Fe-based metal–organic frameworks (Fe-MOFs).

### 4.8. In Situ Diffuse Reflectance Infrared Fourier Transform Spectroscopy (In Situ DRIFTS)

In situ diffuse reflectance infrared Fourier transform spectroscopy (In Situ DRIFTS) was performed on the BRUKER VERTEX 70 spectrometer (Bruker, Bremen, Germany) equipped with a liquid nitrogen-cooled HgCdTe detector. First, 100 mg of catalyst was added to the in situ reaction tank and pre-treated in pure N_2_ at 150 °C for 1 h, and then background data were collected. Then, high-purity CO_2_ gas was passed into the reaction chamber at a speed of 30 mL/min, and the spectrum was collected under continuous light for 60 min. The temperature of the reaction tank is maintained at 25 °C by circulating cooling water.

### 4.9. Evaluation of Photocatalytic CO_2_ Reduction Performance

The photocatalytic CO_2_ reduction reaction was conducted in a fully automated integrated unit (MC-SPB10 PT, Beijing Merry Change Technology Co., Ltd., Beijing, China), which is equipped with a vacuum pump and a cooling water circulator specifically designed for the photocatalytic system. Initially, 100 mg of catalyst was dispersed in a quartz reactor containing a mixture of 100 mL deionized water and 100 μL acetonitrile. The reactor was then secured to the device using high-vacuum sealing grease to ensure the airtightness of the reaction system. Before initiating the reaction, the vacuum pump was activated to evacuate the air from the reaction system until the pressure reached −80 kPa. Subsequently, high-purity CO_2_ (99.999%) was gradually introduced into the system until the pressure stabilized at −30 kPa. This evacuation and pressurization cycle was repeated three times to maximize the removal of residual air from the reaction system. Following this, the mixture was stirred for 10 min to achieve adsorption equilibrium between the catalyst and CO_2_. The light source, a 300 W Xenon lamp (MC-PF300C, Beijing Merry Change Technology Co., Ltd., Beijing, China) with a 420 nm filter, was then turned on to commence the experiment. The reactor temperature was maintained at 6 °C by the circulating water-cooling system. Quantitative analysis was conducted hourly via gas chromatography (GC9790PLUS, Zhejiang Fuli Analytical Instrument Co., Ltd., Taizhou, China) utilizing automated sampling software.

### 4.10. Photoelectrochemical Measurements

An electrochemical workstation (CHI660E, Chenhua Instruments, Shanghai, China) was utilized for the photoelectrochemical measurements. The reference electrode was an Ag/AgCl electrode, the counter electrode was a platinum sheet, and the working electrode was the sample film. The preparation procedure for the sample film involved dispersing 10 mg of catalyst in 2 mL of ethanol solution followed by ultrasonication for 30 min. The resulting homogeneous dispersion was drop-cast onto fluorine-doped tin oxide (FTO) conductive glass to form a thin film, which was subsequently dried in an oven at 80 °C overnight. A 300 W xenon lamp (λ ≥ 420 nm) was utilized as the light source for the photocurrent response measurements. The electrolyte solution for this test was 0.5 mol/L sodium sulfate (Na_2_SO_4_). For the EIS tests, the electrolyte consisted of 0.5 mol/L potassium hexacyanoferrate (III) (K_3_[Fe(CN)_6_]). The LSV tests were conducted using a 0.2 mol/L Na_2_SO_4_ electrolyte. The CV tests employed a mixed electrolyte solution of 0.01 mol/L K_3_[Fe(CN)_6_] and 0.01 mol/L K_4_[Fe(CN)_6_] in a 1:1 ratio.

### 4.11. Synchrotron-Radiation X-Ray Absorption Fine Structure (XAFS) Spectroscopy

XAFS measurements were conducted using a 21A nanodiffraction beamline at the Taiwan Photon Source, National Synchrotron Radiation Research Center. This facility employs a four-reflection channel-cut silicon (111) monochromator to deliver monochromatic X-rays for high-sensitivity experiments. At the beamline’s endstation, a transmission and fluorescence XAFS setup was implemented, featuring three ionization chambers and a Lytle/SDD detector array, positioned downstream of a Kirkpatrick–Baez mirror system for focused beam delivery. Under typical operating conditions, the incident photon flux ranged from 1 × 10^11^ to 3 × 10^10^ photons per second across X-ray energies spanning 6–27 keV.

### 4.12. Positron Annihilation Lifetime Spectral (PALS)

PALS measurements were performed using a coincidence detection system equipped with BaF2 scintillators, achieving a temporal resolution of 230 ps (FWHM). The radioactive 22Na source (activity: 3 μCi) was encapsulated between two symmetrical specimen slices (φ10.0 mm × 1.0 mm) through aluminum foil wrapping to form a sandwich configuration. To ensure measurement reliability, triplicate independent tests were conducted under ambient conditions (25 °C), with each spectrum accumulating 1 × 10^6^ counts.

## Data Availability

The original contributions presented in this study are included in the article/Supplementary Material. Further inquiries can be directed at the corresponding author(s).

## Supplementary Materials

The following supporting information can be downloaded at https://www.mdpi.com/article/10.3390/nano15080618/s1. Figure S1. SEM images of V_O_-BWO (a) and Fe-MOFs (b); Figure S2. XRD patterns of BWO, V_O_-BWO, Fe-MOFs, and 1.0FM/V_O_-BWO; Figure S3. Raman spectra of BWO, V_O_-BWO, Fe-MOFs, and 1.0FM/V_O_-BWO; Figure S4. High-resolution XPS spectra of the core levels of O 1s on V_O_-BWO and 1.0FM/V_O_-BWO; Figure S5. (a) UV–Vis DRS spectra of BWO, V_O_-BWO, Fe-MOFs, 0.5FM/V_O_-BWO, 1.0FM/V_O_-BWO, 2.0FM/V_O_-BWO, 5.0FM/V_O_-BWO, and 10FM/V_O_-BWO. (b) Tauc plot of BWO, V_O_-BWO, and Fe-MOFs; Figure S6. SEM images of 1.0FM/V_O_-BWO (a) after one reaction and (b) after five cycles of reactions; Figure S7. Nitrogen adsorption–desorption isotherms of BWO, V_O_-BWO, Fe-MOFs, and 1.0FM/V_O_-BWO; Figure S8. NH_3_-TPD of 1.0FM/V_O_-BWO; Figure S9. Water contact angle tests of V_O_-BWO (a), Fe-MOFs (b), and 1.0FM/V_O_-BWO (c); Figure S10. In situ DRIFTS spectra of Fe-MOFs in photocatalytic CO_2_ reduction; Table S1. Relative content of elements in 1.0FM/V_O_-BWO; Table S2. Positron lifetime parameters of V_O_-BWO and 1.0FM/V_O_-BWO; Table S3. Summary of the photocatalytic CO_2_ reduction performance of Bi_2_WO_6_- and Fe-MOFs-based catalysts; Table S4. Porous parameters of BWO, V_O_-BWO, Fe-MOFs, and 1.0FM/V_O_-BWO [58,59,60,61,62,63,64].
