# Supplementary material for: Defect-Engineered Z-Scheme Heterojunction of Fe-MOFs/Bi2WO6 for Solar-Driven CO2 Conversion: Synergistic Surface Catalysis and Interfacial Charge Dynamics
Source: Nanomaterials (Basel). 2025 Apr 17;15(8):618. doi: 10.3390/nano15080618 (PMC12029626; doi:10.3390/nano15080618)
Supplement: Supplementary file 1 [file nanomaterials-15-00618-s001.zip › nanomaterials-3569652-supplementary.pdf]

Supporting Information

Defect-Engineered Z-Scheme Heterojunction of Fe-MOFs/Bi_2WO_6 for Solar-Driven CO_2 Conversion: Synergistic Surface Catalysis and Interfacial Charge Dynamics

Ting Liu [1,2,3], Yun Wu [1], Hao Wang [1,2,3,*], Jichang Lu [2,3,4] and Yongming Luo [1,2,3,4,*]

[1] Faculty of Chemical Engineering, Kunming University of Science and Technology, Kunming 650500, China; liuting1@stu.kust.edu.cn (T.L.); 202210801211@stu.kust.edu.cn (Y.W.)
[2] Key Laboratory of Yunnan Province for Synthesizing Sulfur-Containing Fine Chemicals, Kunming 650500, China; lujichangc7@kust.edu.cn
[3] The Innovation Team for Volatile Organic Compounds Pollutants Control and Resource Utilization of Yunnan Province, Kunming 650500, China
[4] Faculty of Environmental Science and Engineering, Kunming University of Science and Technology, Kunming 650500, China
* Correspondence: haowang668@kust.edu.cn (H.W.); environcatalysis@kust.edu.cn (Y.L.)

1. Supplementary Figure

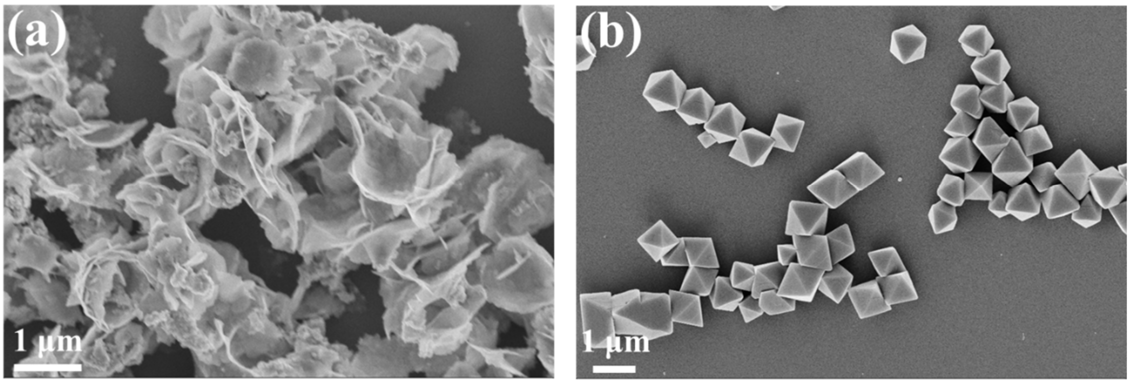

Figure S1. SEM images of V_O-BWO (a) and Fe-MOFs (b).

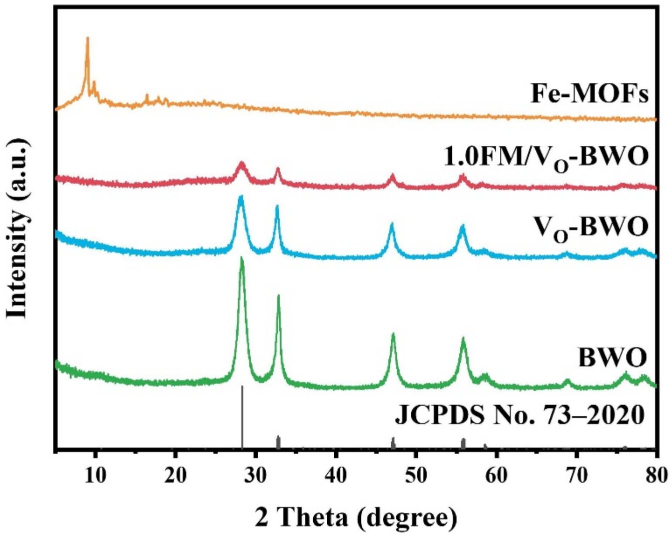

Figure S2. XRD patterns of BWO, V_O-BWO, Fe-MOFs, and 1.0FM/V_O-BWO.

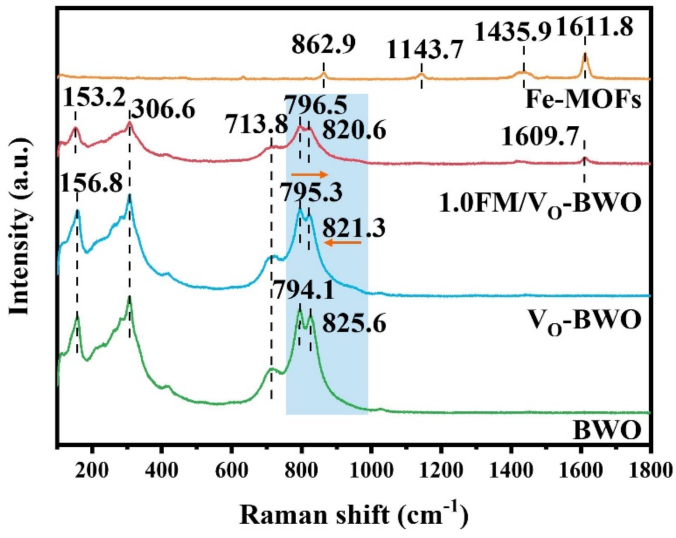

Figure S3. Raman spectra of BWO, VO-BWO, Fe-MOFs, and 1.0FM/VO-BWO.

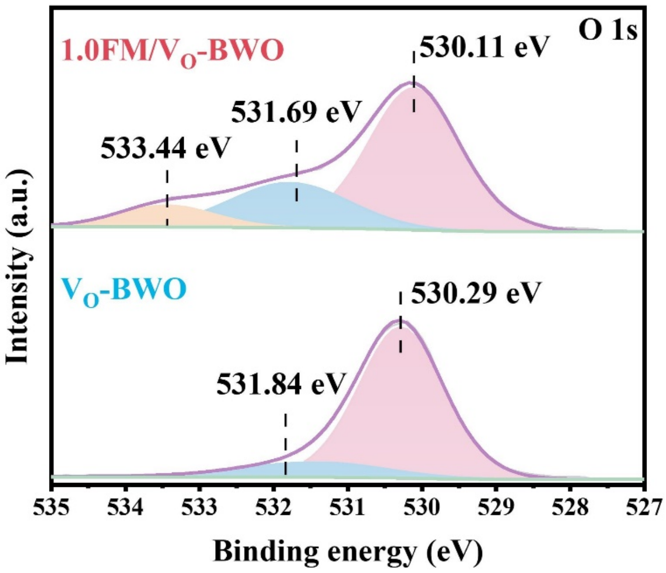

Figure S4. High-resolution XPS spectra of the core levels of O 1s on VO-BWO and 1.0FM/VO-BWO.

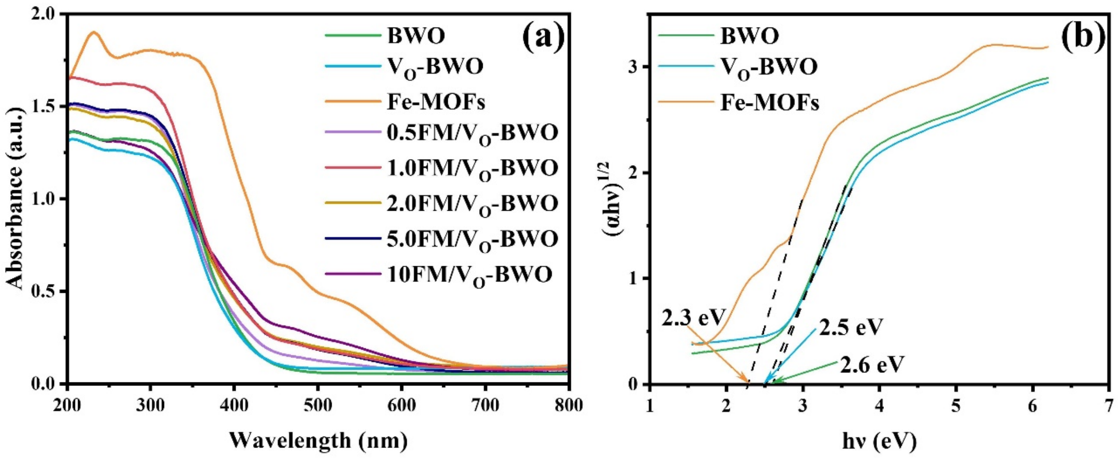

Figure S5. (a) UV–Vis DRS spectra of BWO, V_O-BWO, Fe-MOFs, 0.5FM/V_O-BWO, 1.0FM/V_O-BWO, 2.0FM/V_O-BWO, 5.0FM/V_O-BWO, and 10FM/V_O-BWO. (b) Tauc plot of BWO, V_O-BWO, and Fe-MOFs.

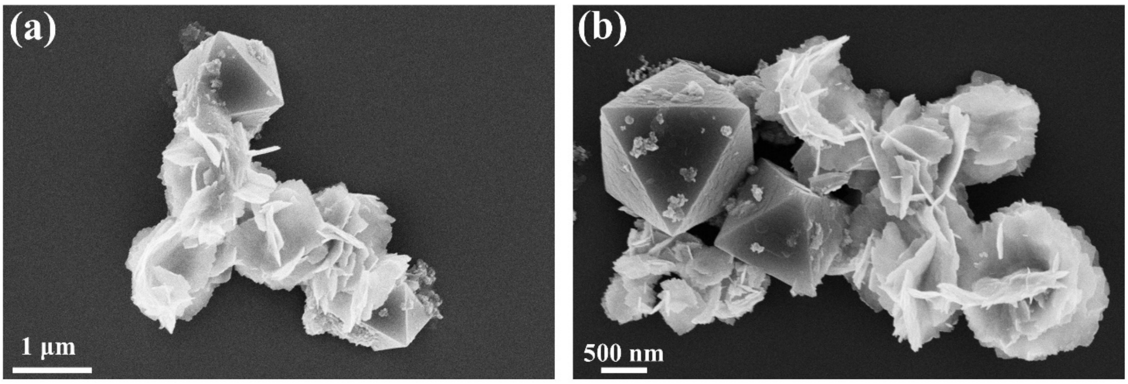

Figure S6. SEM images of 1.0FM/V_O-BWO (a) after one reaction and (b) after five cycles of reactions.

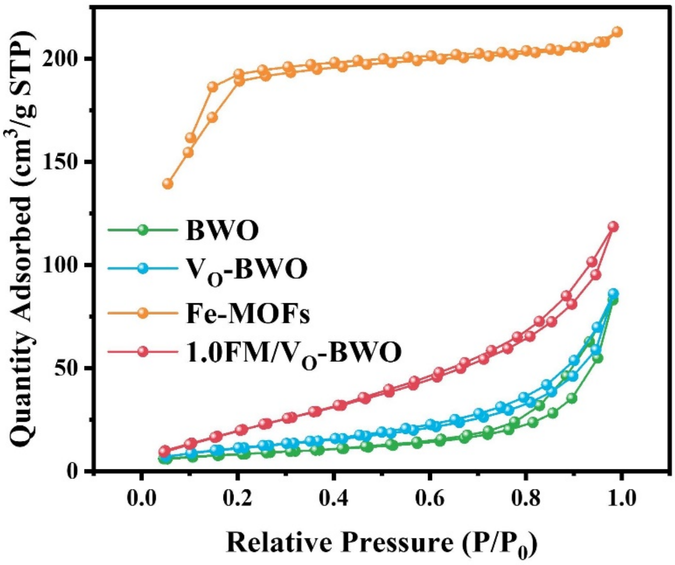

Figure S7. Nitrogen adsorption–desorption isotherms of BWO, V_O-BWO, Fe-MOFs, and 1.0FM/V_O-BWO.

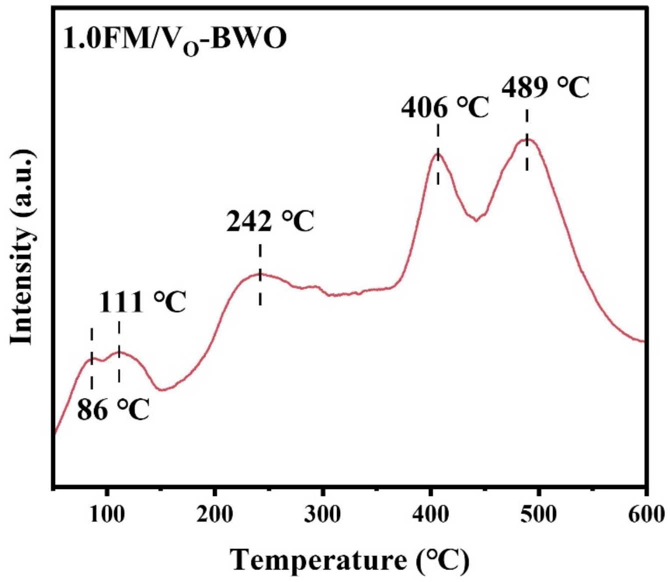

Figure S8. NH_3-TPD of 1.0FM/VO-BWO.

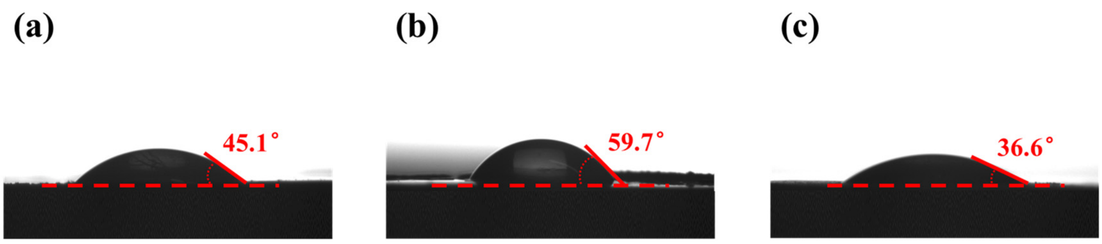

Figure S9. Water contact angle tests of V_O-BWO (a), Fe-MOFs (b), and 1.0FM/V_O-BWO (c).

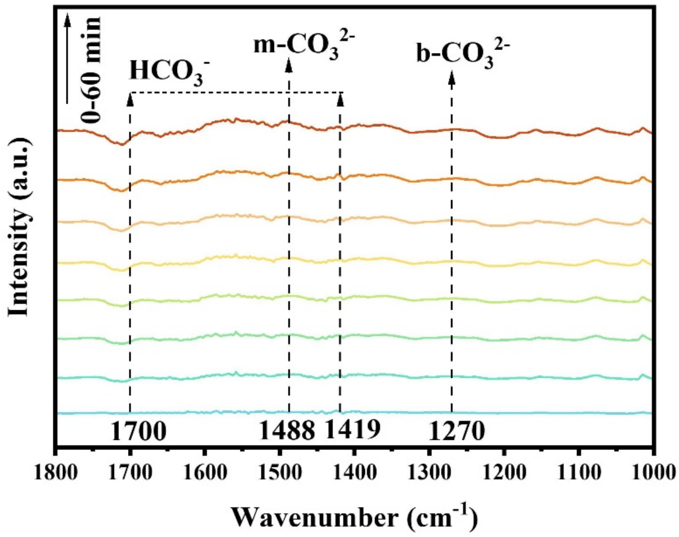

Figure S10. In situ DRIFTS spectra of Fe-MOFs in photocatalytic CO_2 reduction.

2. Supplementary Tables

Table S1. Relative content of elements in 1.0FM/V_O-BWO.

Element	Mass Fraction (%)	Mass Error (%)
**O**	15.94	2.84

**Fe**	1.70	0.20
**W**	23.72	2.12
**Bi**	58.64	5.66

Table S2. Positron lifetime parameters of V_O-BWO and 1.0FM/V_O-BWO.

**Sample**	**τ_1/ps**	**I_1/%**	**τ_2/ps**	**I_2/%**	**τ_3/ns**	**I_3/%**
V_O-BWO	216.3	45.7	317.3	52.3	2.389	2.0
1.0FM/V_O-BWO	221.3	40.3	320.3	57.3	2.632	2.4

Table S3. Summary of the photocatalytic CO_2 reduction performance of Bi_2WO_6- and Fe-MOFs-based catalysts.

**Photocatalyst**	**Condition**	**Light source**	**Products /μmol·g^{-1}·h^{-1}**	**Ref.**
1.0FM/V_O-BWO	H_2O+ACN	> 420 nm	CO, 15.12	This work
0.2 $Cs_2AgBiBr_6/Bi_2WO_6$	EA+IPA	420 ~ 800 nm	CO, 1.85	[58]
10CABB/V_{Bi-O}BWO	H_2O	> 420 nm	CO, 12.3	[35]
0.5 CdS/Bi_2WO_6-S	EA+IPA	420 ~ 800 nm	CO, 6.86	[59]
BWO-C_2	H_2O (vapor)	UV–Vis	CO, 7.12	[28]
$Cs_3Bi_2I_9/Bi_2WO_6$	H_2O (vapor)	UV–Vis	CO,7.33	[60]
NH_2-MIL-53(Fe)	TEOA	400 ~ 780 nm	CO, 3.14	[61]
$MAPbI_3$@PCN-221($Fe_{0.2}$)	EA/CAN+H_2O	> 420 nm	CO, 4.16	[62]
20% CdS/MIL-53 (Fe/Mn)	ethanol	> 420 nm	CO, 2.67	[63]
PSMIL-53(Fe)	TEOA+H_2O	> 420 nm	CO, 11.8	[64]
PSMIL-88A(Fe)	TEOA+H_2O	> 420 nm	CO, 8.8	[64]
PSMIL-88B(Fe)	TEOA+H_2O	> 420 nm	CO, 6.4	[64]

Table S4. Porous parameters of BWO, V_O-BWO, Fe-MOFs, and 1.0FM/V_O-BWO.

**Samples**	**S_{BET} (m^2/g)**	**Total Pore Volume (cm^3/g)**	**Average Pore Size (nm)**
BWO	29.549	0.1285	17.4002
V_O-BWO	42.590	0.1331	12.4961
Fe-MOFs	598.073	0.3294	2.20298
1.0FM/V_O-BWO	88.800	0.1833	8.25905
